# Fat quantification in dual-layer detector spectral CT: How to handle iron overload, varying tube voltage and radiation dose Indices

**DOI:** 10.1371/journal.pone.0302863

**Published:** 2024-05-23

**Authors:** Isabel Molwitz, Graeme Michael Campbell, Tobias Knopp, Niklas Schubert, Jennifer Erley, Anastassia Löser, Gerhard Adam, Jin Yamamura, Roland Fischer, Ann-Kathrin Ozga, Patryk Szwargulski

**Affiliations:** 1 Department of Diagnostic and Interventional Radiology and Nuclear Medicine, University Medical Center Hamburg-Eppendorf, Hamburg, Germany; 2 Philips GmbH Market DACH, Hamburg, Germany; 3 Institute for Biomedical Imaging, Technical University, Hamburg, Germany; 4 Section for Biomedical Imaging, University Medical Center Hamburg-Eppendorf, Hamburg, Germany; 5 Department of Radiotherapy, University Hospital Schleswig-Holstein Campus Lübeck, Lübeck, Germany; 6 Evidia Group, Berlin, Germany; 7 Hematology and Oncology Department, UCSF Benioff Children’s Hospital, Oakland, California, United States of America; 8 Institute of Medical Biometry and Epidemiology, University Medical Center Hamburg-Eppendorf, Hamburg, Germany; Medical University of Vienna, AUSTRIA

## Abstract

**Objectives:**

Opposed to other spectral CT techniques, fat quantification in dual-layer detector CT (dlCT) has only recently been developed. The impact of concomitant iron overload and dlCT-specific protocol settings such as the dose right index (DRI), a measure of image noise and tube current, on dlCT fat quantification was unclear. Further, spectral information became newly available <120 kV. Therefore, this study’s objective was to evaluate the impact of iron, changing tube voltage, and DRI on dlCT fat quantification.

**Material and methods:**

Phantoms with 0 and 8mg/cm^3^ iron; 0 and 5mg/cm^3^ iodine; 0, 10, 20, 35, 50, and 100% fat and liver equivalent, respectively, were scanned with a dlCT (CT7500, Philips, the Netherlands) at 100kV/20DRI, 120kV/20DRI, 140kV/20DRI, and at 120kV/16DRI, 120kV/24DRI.

Material decomposition was done for fat, liver, and iodine (A1); for fat, liver, and iron (A2); and for fat, liver, and combined reference values of iodine and iron (A3). All scans were analyzed with reference values from 120kV/20DRI. For statistics, the intraclass correlation coefficient (ICC) and Bland-Altman analyses were used.

**Results:**

In phantoms with iron and iodine, results were best for A3 with a mean deviation to phantom fat of 1.3±2.6% (ICC 0.999 [95%-confidence interval 0.996–1]). The standard approach A1 yielded a deviation of -2.5±3.0% (0.998[0.994–0.999]), A2 of 6.1±4.8% (0.991[0.974–0.997]). With A3 and changing tube voltage, the maximal difference between quantified fat and the phantom ground truth occurred at 100kV with 4.6±2.1%. Differences between scans were largest between 100kV and 140kV (2.0%[-7.1–11.2]). The maximal difference of changing DRI occurred between 16 and 24 DRI with 0.4%[-2.2–3.0].

**Conclusion:**

For dlCT fat quantification in the presence of iron, material decomposition with combined reference values for iodine and iron delivers the most accurate results. Tube voltage-specific calibration of reference values is advisable while the impact of the DRI on dlCT fat quantification is neglectable.

## 1. Introduction

Fat quantification is relevant to detect hepatic steatosis, characterize potentially suspicious lesions [1], or measure muscle quality for sarcopenia diagnostics [2]. With spectral Computed Tomography (CT) it is feasible to quantify fat in clinical routine CT scans [3]. Opposed to tissue attenuation in Hounsfield units as an indirect measure of fat contents [4], fat quantification in spectral CT is not biased by contrast agent [3]. It allows to measure subtle fat contents and thus to differentiate, e.g., benign adrenal adenomas or pulmonary hamartoma from malignant lesions [5–7]. Compared to MRI fat quantification, CT has faster acquisition times and is easily applicable to critically ill or claustrophobic patients.

There are a variety of spectral CT techniques such as fast-kVp-switching CT, dual-source CT, or dual-layer detector spectral CT (dlCT) [8]. In dlCT it is advantageous, that no prospective selection of a spectral scan mode is necessary and spectral information thus automatically generated with every scan [9]. However, in contrast to the other spectral CT techniques, the feasibility of fat quantification with dlCT has only recently been demonstrated [10]. So far, dlCT has been validated in the context of contrast-phase independent quantification of hepatic steatosis [11] and for the detection of myosteatosis [10].

What had not yet been evaluated, was the impact of dense materials other than iodine, such as iron, on dlCT fat quantification results. This is of clinical relevance, as, e.g., iron overload (> 2 mg Fe/g dry weight) [12] is common in hepatic steatosis and chronic liver disease [13]. Hepatic steatosis as in non-alcoholic fatty liver disease (NAFLD) has a worldwide prevalence about 25% [14]. Among patients with NAFLD alone, about one-third suffers from at least mild hepatic iron overload [15]. Therefore, valid fat quantification in the presence of iron is necessary. While it is known that fat contents are underestimated in the presence of iron in dual-source CT [16,17], the potential impact of iron on dlCT fat quantification and how to deal with it was not clear, yet.

Moreover, besides the scan phase, evaluation of the impact of scan parameter settings on dlCT fat quantification results was pending. This concerns tube voltage as the spectral base images, that contain spectral data, newly became available for scans below 120 kV [18]. Further, the impact of the dlCT vendor-specific scan parameter the so-called dose right index (DRI) was unclear. The DRI defines image noise by tube current (a decrease of the DRI by -1, decreases the tube current by 12% and increases the image noise by 6% and vice versa) [19]. An impact of at least tube voltage was deemed likely as dlCT material decomposition is based on the energy- and material-specific attenuation coefficients that result from the Compton scattering and photoelectric effect [20]. Both, the photoelectric effect, and Compton scattering depend on the X-ray energy, an object’s atomic number (Z), and electron density. Especially, the photoelectric effect has a strong dependence on energy. It is dominant at low energies, and quickly decreases at higher energies. Conversely, Compton scattering is primarily dependent on electron density and has a weaker dependence on the energy spectrum.

Therefore, this study aimed to evaluate the impact of concomitant iron overload, changes in tube voltage and DRI on dlCT fat quantification using phantoms in order to develop recommendations on how to handle these aspects for clinical dlCT fat quantification.

## 2. Material and methods

Due to the study design being exclusively based on phantom scans (no human participants), neither ethics committee approval nor participant consent was required.

### 2.1 Phantoms and measurement set-up

**Fig 1:**
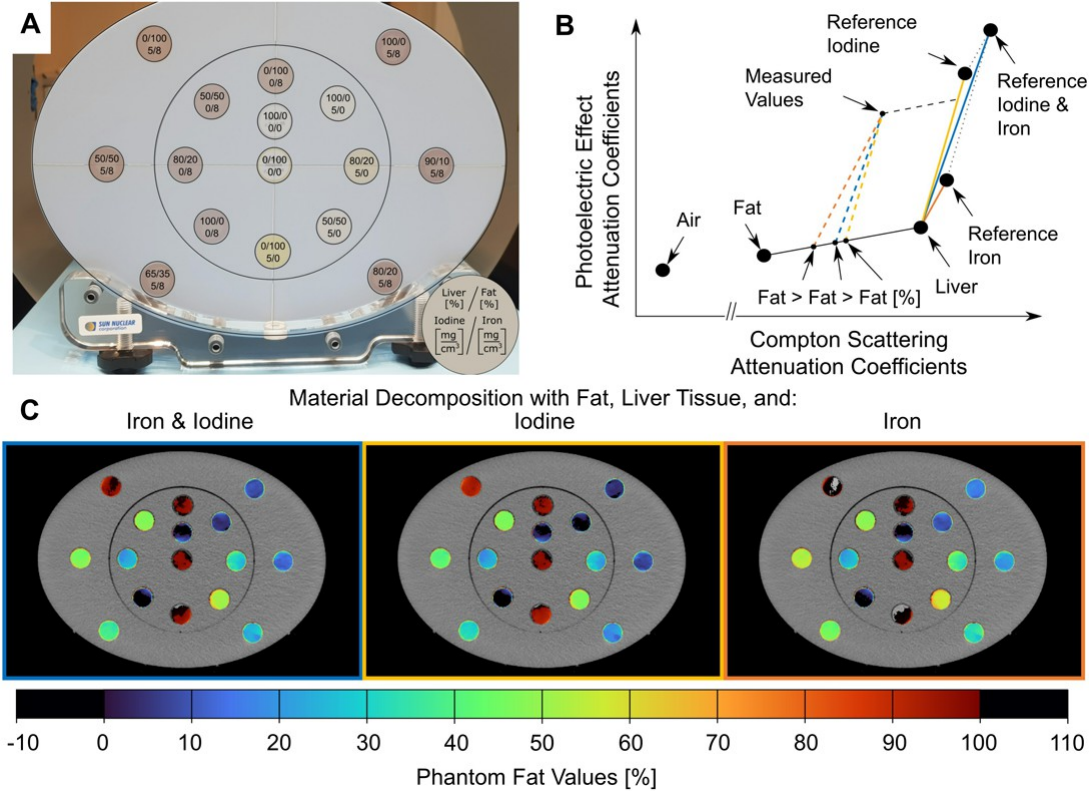
Measurement set-up (a), schematic display of material decomposition (b), and color-coded quantified fat values (c). As can be seen in (b) the reference values for iron (orange line) are slightly to the right compared to that of iodine (yellow line). To include both iodine and iron in the material decomposition a line (blue) was plotted between the iron and iodine slope, which represents calculated combined reference values for iron and iodine. In (c) on the left a material decomposition approach including both iodine and iron, in the middle with iodine only, and on the right with iron only as high-Z materials is displayed. Decomposition with iron only (c, right image) overestimates fat values in all phantom tubes with iodine.

Commercial phantom tubes that match the spectral characteristics of 0 or 5 mg/cm^3^ iodine, 0 or 8 mg/cm^3^ iron, and varying volume fractions of fat and liver tissue (Gammex; Sun Nuclear Corporation, Melbourne, Australia) were inserted into a multi-energy CT phantom (Gammex, Sun Nuclear Corporation, Melborne, Australia). The multi-energy phantom represents human body volumes and water densities (**Fig 1A**). The phantom tube concentrations are listed in detail in **Table 1** and visualized in **Fig 1A**. Please note that the volume fractions of fat and liver tissue refer to the volume not occupied by iodine or iron. All phantom tubes were cylindric and measured 28.5 cm in length and 1.7 cm in diameter.

**Table 1:** Phantom tube concentrations.

Liver Volume Fraction [%]	Fat Volume Fraction [%]	+ Iodine [mg/cm^3^] / Iron [mg/cm^3^]
100	0	0/8; 5/0; 5/8; 0/0
90	10	5/8
80	20	0/8; 5/0; 5/8
65	35	5/8
50	50	0/8; 5/0; 5/8
0	100	0/8; 5/0; 5/8; 0/0

All analyses were performed for cylindric volumes of interest (VOIs), which were defined within the phantom tubes. The minimum size of VOIs was 1.3 cm in diameter and 8.5 cm in length. The utmost slices were excluded from the VOIs, as partial volume averaging occurred at the edges of the phantom tubes.

### 2.2 Scan parameters

The phantom set-up was subjected to scans with either changing tube voltage or changing DRI. In detail, scans (CT7500, Philips, Best, the Netherlands) were performed with 20 DRI and changing tube voltage of 100 kV, 120 kV, and 140 kV. Also, scans were performed with a constant tube voltage of 120 kV and changing DRI of 16, 20, and 24 DRI. Thus, a total of 5 scans were performed (20 DRI/100 kV, 20 DRI/120 kV, 20 DRI/140 kV; 16 DRI/120 kV, 24 DRI/120 kV).

The DRI is the vendor-specific protocol setting used to define the mAs per patient size, while maintaining a targeted consistent image quality within a single acquisition and between patients. For any given DRI, the mAs is modulated to account for the variations in patient body attenuation compared to a predefined reference dose for a reference water equivalent diameter of 29 cm. Decreasing (or increasing) the DRI by 1 unit will decrease (or increase) the average tube current by 12% and increase (or decrease) the image noise by 6%.

Collimation was 128 x 0.625 mm and the matrix 512 x 512. The pitch was 1 and the table speed 200 mm/s. The reconstructed slice thickness was 5 mm. The resulting voxel size was 0.98 x 0.98 x 5 mm^3^. Scan and reconstruction parameters were identical for each scan.

### 2.3. Material decomposition

Differences in fat quantification were evaluated using distinct material decomposition approaches. In dlCT material decomposition is based on the Compton scattering and photoelectric effect approximations, which for each scanned voxel were extracted from the spectral DICOMs. Briefly, it is based on solving the linear system ***Ax* = *b***, where **x** is the result of the decomposition. Here, ***A*** ∈ ℝ^3×3^ contains the material-specific attenuation coefficients of the Compton scattering and photoelectric effect of three base materials (fat, liver, iodine/iron) and an additional volume conservation constraint (that the sum of the respective values equals 1). ***b*** ∈ ℝ^3^contains the measured photoelectric effect and Compton scattering values of the given voxel [8,10]. For the decomposition, it is thus necessary to know the material-specific attenuation coefficients of the Compton scattering and photoelectric effect of the materials in question. These reference values were extracted from the phantom tubes of the scan with 120 kV/20 DRI, as this was closest to the standard clinical protocol parameters in our department (abdomen or thorax/abdomen: 120 kV, 18 DRI +3 DRI liver boost). As the described material decomposition approach assumes three different materials within one voxel, but some phantom tubes contained four materials (fat equivalent, liver equivalent, iodine, and iron), an additional reference value was calculated for the combined coefficients of iodine and iron.

To evaluate how to best handle coexisting iron, we tested three different material combinations for the material decomposition. In the first approach (A1) we included reference values of fat, liver tissue, and iodine, which would be the standard material combination for hepatic fat quantification (**Fig 1B**, yellow vector). The second approach (A2) was based on reference values of fat, liver tissue, and iron (**Fig 1B**, orange vector). For the third approach (A3) we used reference values of fat, liver tissue, and the calculated mixed reference value for the combined coefficients of iodine and iron (**Fig 1B**, blue vector).

To evaluate the impact of the tube voltage on the material decomposition results, reference values from the scan at 120 kV and 20 DRI were used to quantify fat in scans performed with 100 kV, 120 kV, and 140 kV (each with 20 DRI), respectively.

To assess the impact of the DRI, reference values from the scan at 120 kV/20 DRI were employed to quantify fat at the scans with 16 DRI, 20 DRI, and 24 DRI (each at 120 kV), respectively.

### 2.4. Statistics

Data was summarized by the mean and standard deviation. Analyses were based on the mean fat quantification result from all voxels within a respective VOI. For each phantom tube, fat quantification results for the different material decomposition approaches (A1, A2, A3) were compared to the phantom ground truth by one-way random intraclass correlation coefficients (ICC). Agreement between fat quantification results of scans with fixed DRI but varying tube voltage (all quantified with reference values from 120 kV) were displayed by Bland-Altman plots. Similarly, the agreement between fat quantification results of scans with fixed tube voltage but varying DRI (all quantified with material decomposition reference values from 20 DRI) was displayed by Bland-Altman plots. Due to the very high agreement between the latter, the ICC was additionally employed to further test for agreement. There were no missing data. SPSS (Version 26.0, IBM Corp., Armonk, NY, USA) was employed for statistics.

## 3. Results

### 3.1. Impact of iron on fat quantification results

The impact of iron on fat quantification results was evaluated by comparison of the different material decomposition approaches A1, A2, and A3 for the scan at 120 kV/20 DRI as it was the protocol setting closest to clinical routine.

Taking all phantom tubes into account, the agreement between quantified fat values and phantom fat values was excellent no matter which high-atomic number (high-Z) material was included in the decomposition: A1: iodine, A2: iron, or A3: the combined reference value for iodine and iron. However, the best results were achieved by using the combined reference for iodine plus iron (A3) with an ICC of 0.999 [95%-confidence interval (95%-CI) 0.996–1]. The mean deviation to phantom ground truth for this approach was 1.3% with a standard deviation (SD) of 2.6%. Results for the material decomposition approach A1 (high-Z: iodine) were also good (ICC 0.998 [95%-CI 0.994–0.999]) with a mean deviation to the phantom ground truth of -2.5% SD 3.0%. Using A2 (high-Z: iron) resulted in a smaller but still excellent ICC (0.991 [95%-CI 0.974–0.997]). Correspondingly, for A2 the mean deviation to the phantom ground truth was larger with 6.1% SD 4.8%. In detail, all fat quantification results are provided in the **S1 Table**.

**Fig 2:**
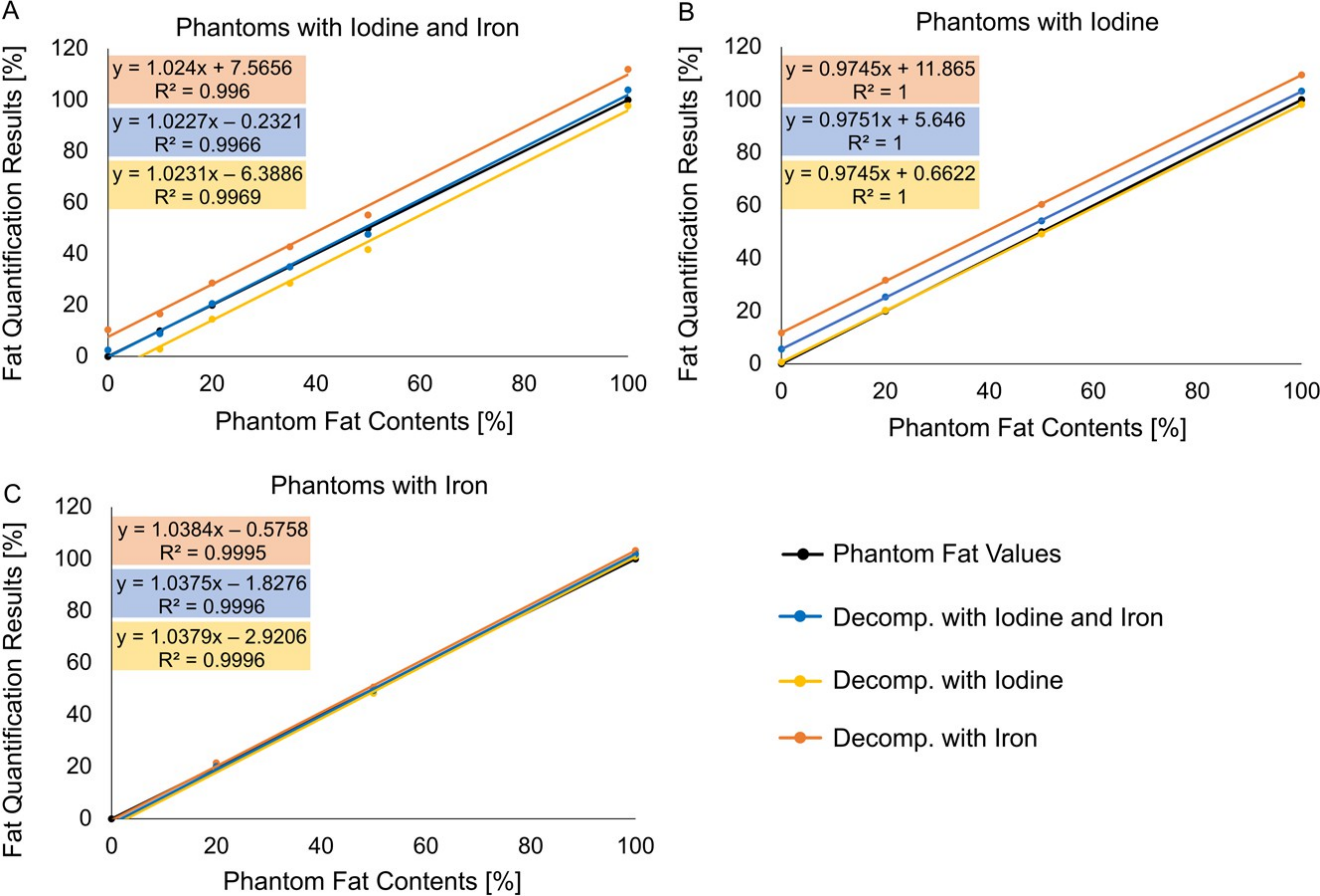
Fat quantification results for different material decomposition approaches in phantoms with iodine and iron (a), iodine (b), and iron (c). In phantom tubes that contained both iodine and iron (a) material decomposition (decomp.) with the combined reference values for iodine and iron (blue) as high-Z material yielded the best results. In phantom tubes that contained only iodine as high-Z material (b) material decomposition with the combined reference for iodine and iron (blue) resulted in slightly higher fat contents than the phantom ground truth (but <5% difference). In phantom tubes that contained only iron as high-Z material (c) all three fat quantification approaches matched phantom fat contents.

Next, phantom tubes were grouped depending on whether they contained both iodine and iron as high-Z material, iodine only, or iron only. Deviation of fat quantification results to the phantom ground truth is displayed for each material decomposition approach (A1, A2, A3) in **Fig 2**. In phantom tubes that contained both iodine and iron (**Fig 2A**), using the material decomposition approach A3 (high-Z: combined reference for iodine and iron) yielded excellent results. With A3, the deviation of quantified fat values to the phantom ground truth was small (0.6%, SD 2.3%). Decomposition with A1 (high-Z: iodine) underestimated phantom fat contents (mean difference -5.6% SD 2.2.%). Decomposition with A2 (high-Z: iron), resulted in fat contents above the ground truth (mean difference 8.4% SD 2.5%) (**Fig 2A**).

In phantoms that contained only iodine as dense material (**Fig 2B**), decomposition with A1 (high-Z: iodine) was best suited with a deviation of quantified fat values to the phantom ground truth of -0.4% SD 1.1% (**Table 2**). Decomposition approach A3 (high-Z: combined reference for iodine and iron) resulted in a slight overestimation (4.6% SD 1.1%) and decomposition with A2 (high-Z: iron) in a large overestimation (10.8% SD 1.1%) (**Figs 1C and 2B**).

**Table 2:** Deviation of fat quantification results compared to the phantom ground truth for each decomposition approach and phantom group.

High-Z Material in Phantom Tube	Material Decomposition with Liver Equivalent, Fat Equivalent, and …	Mean Difference to Phantom Fat Value [%]	Standard Deviation [%]
Iodine and Iron	Iodine and Iron	0.6	2.3
	Iodine	-5.6	2.2
	Iron	8.4	2.5
Iodine only	Iodine and Iron	4.6	1.1
	Iodine	-0.4	1.1
	Iron	10.8	1.1
Iron only	Iodine and Iron	-0.2	1.9
	Iodine	-1.3	1.9
	Iron	1.1	1.9

For phantom tubes that contained only iron as dense material, the results of all three decomposition approaches matched phantom fat values (**Fig 2C**). In detail, all results are listed in **Table 2**.

### 3.2. Impact of changing tube voltage on fat quantification results

The impact of changing tube voltage on fat quantification results was evaluated across all phantom groups. Therefore, the decomposition approach A3 (high-Z: combined reference for iodine and iron), was used, as its results were closest to the phantom ground truth for tubes with iodine and iron, tubes with iron only, and second best for tubes with iodine only (see 3.1). All reference values were generated from the scan at 120 kV/20 DRI, which is closest to the standard clinical protocol.

**Fig 3:**
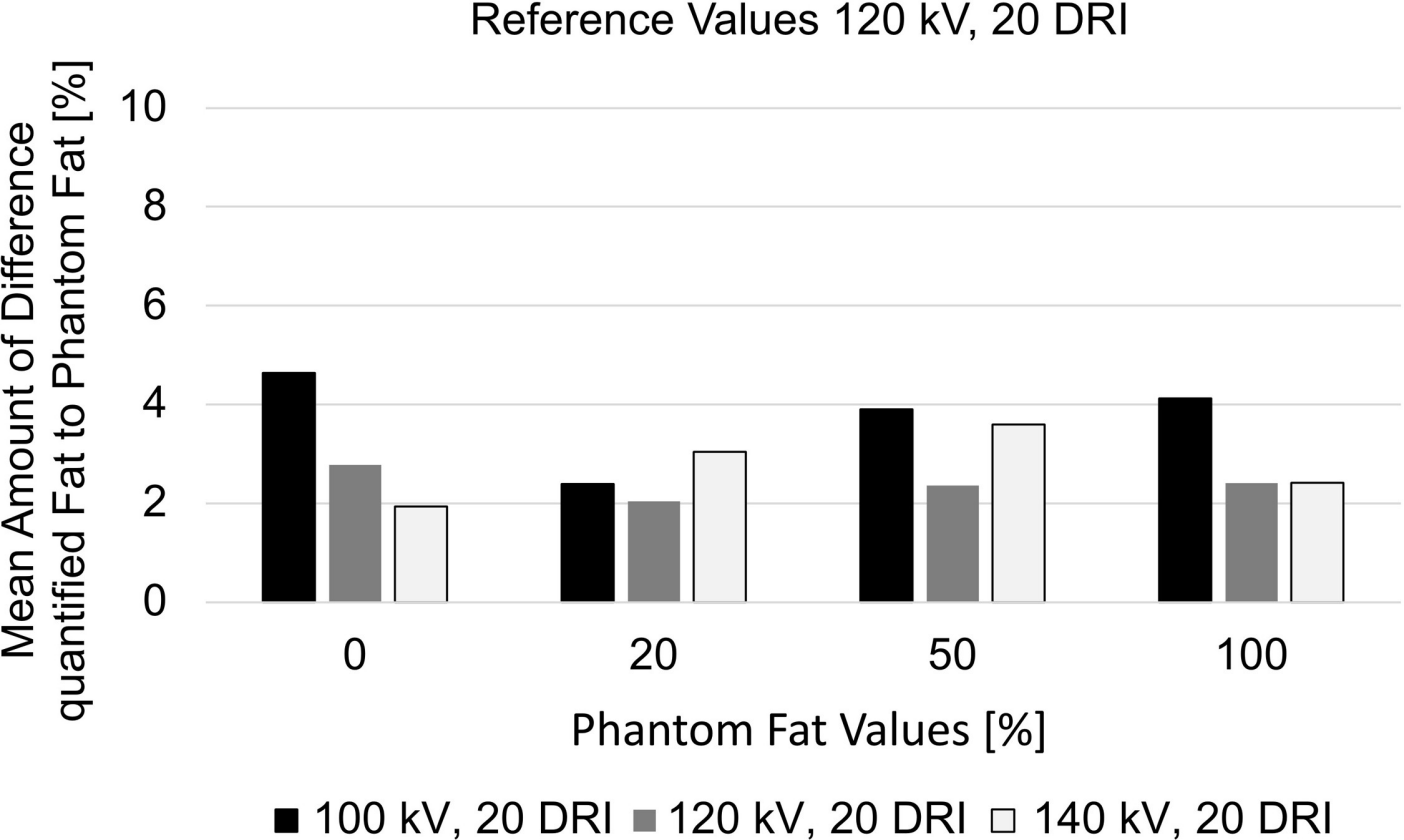
Difference of quantified to phantom fat at 100 kV, 120 kV, and 140 kV. All scans were quantified with material decomposition reference values generated from the scan at 120 kV. The largest differences to the phantom ground truth were found for the scan at 100 kV. Phantom tubes with 10% and 35% fat are not displayed, because they were only present once within the measurement set-up. Abbreviation: DRI: dose right index.

When applying material decomposition A3 with reference values from 120 kV to the scan data at 120 kV, the difference between quantified fat contents and the phantom ground truth was small (**Fig 3**). The largest deviations to the phantom ground truth occurred when employing reference values from 120 kV to the scan data at 100 kV with up to 4.6% SD 2.1% mean difference for the phantom tubes with zero percent fat (**Fig 3**).

For material decomposition with reference values from 120 kV applied to the scan data from 140 kV, the maximum deviation to the phantom ground truth was a mean of 3.6% SD 1.4% for tubes with 50% fat. Information on all deviations is listed in **Table 3**.

**Table 3:** Difference between fat quantification results and phantom ground truth for scans at varying tube voltage when using reference values from 120 kV/20 DRI for material decomposition.

Phantom Fat Content [%]	Mean of the Amount of Difference between Quantified Fat Values and the Phantom Fat Content					
	100 kV, 20 DRI		120 kV, 20 DRI		140 kV, 20 DRI	
	%	SD	%	SD	%	SD
0	4.6	2.1	2.8	2.1	1.9	1.4
20	2.4	0.9	2.0	2.8	3.0	1.2
50	3.9	3.2	2.4	1.8	3.6	1.4
100	4.1	2.6	2.4	1.5	2.4	2.0

*The phantom tubes of 10% and 35% fat contents are not included*, *as there were no multiple observations*.

*Abbreviations: SD standard deviation*.

**Fig 4:**
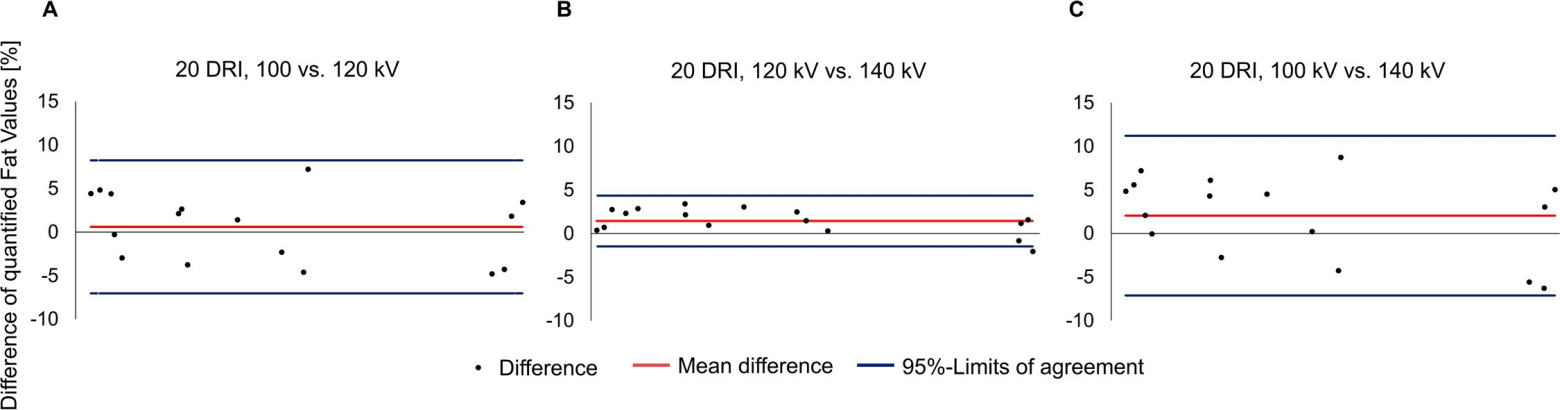
Bland-Altman plots of fat quantification results between 100 kV, 120 kV, and 140 kV. Fat quantification of all scans was performed with material decomposition reference values generated from the scan at 120 kV. The mean difference was 0.6% (95%-limits of agreement -7.0, 8.2) between 100 kV and 120 kV (a), 1.4% (-1.5, 4.4) between 120 kV and 140 kV (b), and the largest between 100 kV and 140 kV (c) with 2.0% (-7.1, 11.2).

Further, the agreement of fat quantification results was evaluated between the scans at 100 kV, 120 kV, and 140 kV when all were analyzed with material decomposition A3 and reference values from 120 kV. It was found to be best between 120 kV and 140 kV with a mean difference of 1.4% in the Bland-Altman (95%-limits of agreement -1.5, 4.4) (**Fig 4**). Between 100 kV and 140 kV agreement was less good with a mean difference of 2.0% and larger 95%-limits of agreement (-7.1, 11.2) (**Fig 4**).

### 3.3. Impact of changing DRI on fat quantification results

The impact of changing DRI on fat quantification results was evaluated across all phantom groups. Thus, the decomposition approach A3 (high-Z: combined reference for iodine and iron) was used, as its results were closest to the phantom ground truth for tubes with iodine and iron, tubes with iron only, and second best for tubes with iodine only (see 3.1). Again, reference values were employed from the scan at 20 DRI/120 kV as the clinical standard protocol.

When applying these for fat quantification from scan data at 16, 20, or 24 DRI (all 120 kV) agreement to the phantom ground truth was always good. The maximum mean of the amount of difference to the phantom ground truth was 2.7% SD 2.1% found for the scan with 20 DRI in the phantom tubes with 0%.

**Fig 5:**
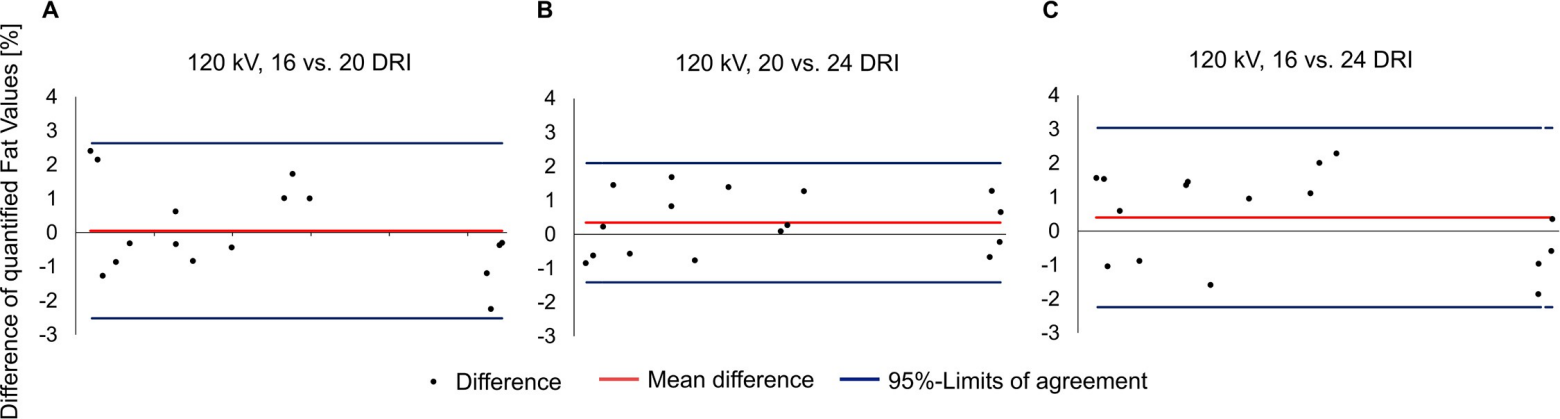
Bland-Altman plots of quantified fat values between 16 DRI, 20 DRI, and 24 DRI. Fat quantification of all scans was performed with material decomposition reference values generated from the scan with a dose right index (DRI) of 20. Overall agreement was high. The mean difference was smallest between 16 and 20 DRI (a), when taking the 95%-limits of agreement into account, agreement was better between 20 and 24 DRI (b). The largest mean difference was found between 16 and 24 DRI (c) (0.4% (95%-limits of agreement -2.2, 3.0).

Concerning agreement between fat quantification results of the scans with varying DRI, when all were analyzed with material decomposition A3 and reference values from scan data at 20 DRI, the mean differences in the Bland-Altman plots were small (**Fig 5**). Between 16 DRI and 20 DRI the mean difference was 0.1% (95%-limits of agreement -2.5, 2.6%), between 20 and 24 DRI it was 0.3% (95%-CI -1.4, 2.1). Between 16 and 24 DRI it was 0.4% (95%-CI -2.2, 3.0). The ICC for 16 to 20 DRI as well as for 16 to 24 was 1 [95%-CI 0.999–1]. For 20 to 24 DRI it was 1 [95%-CI 1–1].

## 4. Discussion

This phantom study investigated the impact of iron, changes in tube voltage and DRI on dlCT fat quantification in order to develop recommendations on how to handle these parameters.

The main findings are that a) material decomposition with a combined reference value for iodine and iron as high-Z material is the best approach to quantify fat in phantoms with iodine and coexisting iron but is also suited if only iodine or only iron is present; and that b) the impact of tube voltage on fat quantification results is larger than that of the DRI. Thus, calibration of material decomposition reference values for tube voltage is advisable while changes in DRI are neglectable.

As this is the first study on the influence of these parameters on dlCT fat quantification our results are discussed with reference to studies on other spectral CT scanner types.

Concerning fat quantification in the presence of iron, Fischer et al. used virtual non-iron maps based on material decomposition with liver tissue, fat, and iodine plus iron (similar to our approach A3) to measure CT numbers in iron-containing fat phantoms [17]. With this approach, they found only minor differences in CT numbers between phantoms with and without iodine. This is in good agreement with our study’s findings for the decomposition approach A3 and phantom tubes with iodine and iron or iron only (**Fig 2A and 2C**).

Comparison to most other studies is hindered as details on the calibration of material decomposition components are frequently missing. For example, one study describes using vendor-specific fat quantification software for dual-source CT, which separates soft tissue, fat, and iodine and then a second readily provided virtual non-iron map based on material decomposition for iron, water, and air [21]. It does, however, not provide details on how the algorithms’ reference values were generated. Further, as the authors state themselves, it seems likely that the algorithms did not strictly separate between iodine and iron, as it was found that iron values within the same patients significantly differed between scan phases. This supports an approach as used in this study to directly employ combined reference values for iodine and iron.

While we had already expected the decomposition approach with iodine and iron to be suitable for VOIs with both materials, it was interesting to note that in phantoms that only contained iodine, the combined decomposition approach still yielded results within 5% compared to the phantom ground truth. For clinical applications such as fat detection in adrenal lesions, tumors, or fat quantification of the skeletal muscle as a parameter of muscle quality [2] for sarcopenia diagnostics [22], a deviation below 5% may still be acceptable. However, concerning hepatic steatosis 5% liver fat is the cut-off between healthy liver tissue and hepatic steatosis [23]. A slight overestimation of fat contents, occurring when using the combined iodine and iron decomposition approach, could thus become relevant in this context.

This study’s findings are of special clinical relevance for patients with liver diseases. Iron overload is common not only in hereditary hemochromatosis, but also in NAFLD, alcoholic fatty liver disease, or hepatitis C infection [13]. Also, in all chronic liver diseases, the reduced synthetic function of the liver results in decreased hepcidin levels [13]. Hepcidin inhibits ferroportin and thereby dietary iron intake. Thus, reduced hepcidin levels cause iron overload [13]. The aforementioned cohorts frequently receive CT scans, e.g., to screen for pre-cirrhotic liver fibrosis, liver cirrhosis, or hepatocellular carcinoma. Using dlCT fat quantification will provide more specific information on the incidence and grade of steatosis than, e.g., ultrasound could do [24]. This is beneficial as valid detection of steatosis allows early treatment to prevent the progress of cirrhosis. As demonstrated by the findings of this study, using only iodine as high-Z material in such patients with iron overload would underestimate fat contents. It is thus advisable to employ the combined decomposition approach with iodine and iron for clinical dlCT fat quantification in these cohorts.

For phantom tubes containing only iron but no iodine, the choice of the high-Z material (iodine, iron, both) did not matter (**Fig 2C**). The maximum difference to the phantom ground truth was 1.3%. Indeed, decomposition with iodine yielded an even smaller deviation (0.2%, SD 1.2%) than that with iron (1.1%, SD 1.2%). These discrepancies may be explained because, for the concentrations used in this experiment, the photoelectric attenuation of iron is much lower than that of iodine (**Fig 1B**). As such, for the phantom tubes containing only iron, altering the vector of the high-Z component will result in relatively small changes along the liver-fat line and therefore small changes in calculated fat content. This indicates that the choice of high-Z material for the decomposition may not be critical in patients with iron overload and non-contrast-enhanced CT scans.

Concerning tube voltage, this study was the first to show the feasibility of dlCT fat quantification below 120 kV. This is of clinical relevance, as according to the indication or tube voltage may differ. Our results demonstrated the bias in fat quantification results when employing once-generated reference values from a standard clinical protocol of 120 kV to scans with changed voltage. For clinical dlCT fat quantification it is thus advisable to generate specific reference values for each respective tube voltage. The impact of tube voltage becomes even more relevant at smaller kV as demonstrated by the largest deviations that were found for the scan at 100 kV. Likely, this is due to the stronger photoelectric effect at lower energies and its relation to high-Z materials (e.g., iodine). Thus, our findings are especially relevant for clinical scenarios with CT scans at low tube voltage, e.g., in pediatric protocols.

On the contrary, the impact of employing reference values from 20 DRI for decomposition of scan data generated with 16 or 24 DRI was negligible. Calibrating new reference values for scans with changing DRI is thus likely not necessary. Again, this is of high clinical relevance as DRI values typically vary in abdominal scans with higher DRIs at the height of the liver (liver boost). Based on this study’s results, when liver fat quantification is prospectively implemented into the dlCT scanner software, it will deliver reliable results without adaption for the liver boost.

A potential limitation of this study is the iron concentrations in the phantom tubes of 8 mg/cm^3^, which is above physiological iron levels (up to 2 mg Fe/g dry weight) [12]. However, that represents well pathological iron concentrations, e.g., in patients with hemochromatosis–a genetic disorder of iron excretion from the liver–who can suffer from iron overload up to 10 times above the usual upper limit (approximately 20 mg/g dry weight) [12]. Some quantified fat values were slightly negative or above 100%. This is a known problem of all material decompositions due to the estimation of values with linear equation systems and could be handled by setting upper and lower limits for the final software output in the clinical routine.

Concerning generalizability, this study’s results on the combined iron and iodine material decomposition approach for fat quantification in the presence of iron should be applicable to other types of spectral CT scanners such as dual-source, or fast-kVp-switching CT, as well.

In conclusion, this study demonstrated that a material decomposition approach for liver equivalent, fat equivalent, and combined iodine and iron reference values delivers the best results for dlCT fat quantification in the presence of iron. This is of clinical relevance for patients with hepatic steatosis and iron overload. Further, dlCT fat quantification is feasible not only as established at 120 kV and 140 kV, but at 100 kV, as well. For scans at different tube voltages, respective calibration of the material decomposition reference values is advisable. Fat quantification results are stable with changing DRI, thus no calibration of reference values for the DRI seems necessary.

## Supporting information

S1 Table Fat quantification results for each phantom tube, scan setting, and material decomposition approach. (DOCX)

S2 Table Detailed description of the fat quantification workflow. (DOCX)
