# Supplementary material for: Fat quantification in dual-layer detector spectral CT: How to handle iron overload, varying tube voltage and radiation dose Indices
Source: PLoS One. 2024 May 23;19(5):e0302863. doi: 10.1371/journal.pone.0302863 (PMC11115214; doi:10.1371/journal.pone.0302863)
Supplement: S1 Table — (DOCX) [file pone.0302863.s001.docx]

**Supplement, Table 1** Fat quantification results for each phantom tube, scan setting, and material decomposition approach

| **Scan Parameter** | | **120 kV, 20 DRI** | | | **100 kV, 20 DRI** | **140 kV, 20 DRI** | **120 kV, 16 DRI** | **120 kV, 24 DRI** |
| --- | --- | --- | --- | --- | --- | --- | --- | --- |
|  | | **Fat Quantification Results [%] based on Material Decomposition with Fat Equivalent, Liver Equivalent, and…** | | | | | | |
| **High Z Material in Phantom Tubes** | **Phantom Tube Fat Content [%]** | **Iodine & Iron, Reference Values**  **120 kV, 20 DRI** | **Iodine,**  **Reference Values**  **120 kV, 20 DRI** | **Iron,**  **Reference Values**  **120 kV, 20 DRI** | **Iodine and Iron, Reference Values**  **120 kV, 20 DRI** | **Iodine and Iron, Reference Values**  **120 kV, 20 DRI** | **Iodine and Iron, Reference Values**  **120 kV, 20 DRI** | **Iodine and Iron, Reference Values**  **120 kV, 20 DRI** |
| **Iodine & Iron** | 0 | 2.5 | -3.8 | 10.4 | 6.9 | -0.3 | 1.2 | 2.2 |
|  | 10 | 8.9 | 2.9 | 16.6 | 5.9 | 6.0 | 8.6 | 9.4 |
|  | 20 | 20.7 | 14.5 | 28.7 | 23.3 | 17.2 | 20.3 | 19.0 |
|  | 35 | 35.0 | 28.6 | 42.8 | 36.4 | 31.9 | 34.5 | 33.6 |
|  | 50 | 47.7 | 41.7 | 55.3 | 45.4 | 45.2 | 48.7 | 47.6 |
|  | 100 | 103.9 | 97.8 | 113.0 | 107.3 | 102.3 | 103.6 | 103.2 |
| **Iodine** | 0 | 5.6 | 0.7 | 11.8 | 5.4 | 3.3 | 4.8 | 4.2 |
|  | 20 | 25.3 | 20.2 | 31.6 | 21.6 | 24.3 | 24.5 | 26.1 |
|  | 50 | 54.2 | 49.1 | 60.4 | 49.6 | 53.9 | 55.2 | 52.9 |
|  | 100 | 103.3 | 98.2 | 109.4 | 99.0 | 105.3 | 102.9 | 103.5 |
| **Iron** | 0 | -2.5 | -3.5 | -1.4 | 2.0 | -2.9 | -0.1 | -1.6 |
|  | 20 | 20.1 | 19.0 | 21.6 | 22.3 | 18.0 | 20.8 | 19.3 |
|  | 50 | 49.4 | 48.3 | 50.8 | 56.6 | 47.9 | 51.2 | 49.1 |
|  | 100 | 102.0 | 101.0 | 103.3 | 103.9 | 100.8 | 99.8 | 100.7 |
| **None** | 0 | -0.5 | -0.4 | -0.5 | 4.3 | -1.2 | 1.6 | 0.1 |
|  | 100 | 100.5 | 100.4 | 100.4 | 95.7 | 101.3 | 99.3 | 101.1 |
