# Supplementary material for: Fat quantification in dual-layer detector spectral CT: How to handle iron overload, varying tube voltage and radiation dose Indices
Source: PLoS One. 2024 May 23;19(5):e0302863. doi: 10.1371/journal.pone.0302863 (PMC11115214; doi:10.1371/journal.pone.0302863)
Supplement: S2 Table — (DOCX) [file pone.0302863.s002.docx]

**Description of the Fat Quantification Workflow**

From the spectral base images (SBI) conventional, photoelectric effect, and Compton scattering images were generated. These images depict the total, photoelectric effect, and Compton scattering attenuation value in each voxel, respectively. In ITK-SNAP [1] regions of interest (ROI) were defined within the conventional images and transferred to the photoelectric effect and Compton scattering images. For the exact ROI definition within the phantom tubes please refer to the method section of this article.

The photoelectric effect and Compton scattering value per voxel within each defined ROI were then used for material quantification using a self-programmed Julia script [2]. This script performed material quantification by solving the linear system of equations:

Eq. 1 ${x =A}^{-1}b=x$

where $b=\left( C_{voxel}P_{voxel}1 \right)^{T}\in R^{3}$

and $A=\left( \begin{matrix} C_{fat} & C_{iodine/iron} & C_{liver} \\ P_{fat} & P_{iodine/rion} & P_{liver} \\ 1 & 1 & 1 \end{matrix} \right)\in R^{3\times3}$.

Therefore, it was necessary to know the reference attenuation values of the photoelectric effect and Compton scattering $P_{voxel}$ and $C_{voxel}$ of all materials in question. These were determined from the scan at 120 kV with a dose right index of 20 as follows: For fat, measurement values of the phantom tube with 100% fat, 0% liver, 0 mg/cm^3^ iodine and 0 mg/cm^3^ iron, respectively were employed. For liver, the corresponding 100% liver, 0% fat, and 0 mg/cm^3^ iodine, 0 mg/cm^3^ iron tube was used. For iron, reference values were generated from the tubes with 100% liver tissue, 0% fat, 0 mg/cm^3^ iodine, and 8 mg/cm^3^ iron. For iodine, reference values were generated from the tubes with 100% liver tissue, 0% fat, 0 mg/cm^3^ iron, and 5 mg/cm^3^ iodine. Combined reference values for phantoms that contained both, iodine and iron, were generated from the tubes with 100% liver, 0% fat, 5 mg/cm^3^ iodine, and 8 mg/cm^3^ iron.

Calibration lines were not necessary as the material decomposition approach is inherently based on the assumption of linear development of the attenuation values of all materials. Thus, the reference values were sufficient.

The result of the decomposition was then given by calculating ${x=A}^{-1}b$,

where $x=\left( x_{fat}x_{iodine/iron}x_{liver} \right)^{T}\in R^{3}$.

Based on the fat quantification results of all voxels within one ROI, a mean fat value with standard deviation was calculated.

**Literature**

1. Yushkevich PA, Piven J, Hazlett HC, Smith RG, Ho S, Gee JC, et al. User-guided 3D active contour segmentation of anatomical structures: significantly improved efficiency and reliability. Neuroimage. 2006;31(3):1116-28. Epub 2006/03/21. doi: 10.1016/j.neuroimage.2006.01.015. PubMed PMID: 16545965.

2. Bezanson J, Edelman A, Karpinski S, Shah VB. Julia: a fresh approach to numerical computing. SIAM Rev. 2017;59(1):65–98. doi: 10.1137/141000671.
